# Toward an Integrative Computational Model of the Guinea Pig Cardiac Myocyte

**DOI:** 10.3389/fphys.2012.00244

**Published:** 2012-07-05

**Authors:** Laura Doyle Gauthier, Joseph L. Greenstein, Raimond L. Winslow

**Affiliations:** ^1^Department of Biomedical Engineering, Institute for Computational Medicine, The Johns Hopkins University School of Medicine and Whiting School of EngineeringBaltimore, MD, USA

**Keywords:** calcium cycling, calcium-induced calcium-release, cardiac myocyte, computational model, excitation-contraction coupling, mitochondrial energetics

## Abstract

The local control theory of excitation-contraction (EC) coupling asserts that regulation of calcium (Ca^2+^) release occurs at the nanodomain level, where openings of single L-type Ca^2+^ channels (LCCs) trigger openings of small clusters of ryanodine receptors (RyRs) co-localized within the dyad. A consequence of local control is that the whole-cell Ca^2+^ transient is a smooth continuous function of influx of Ca^2+^ through LCCs. While this so-called graded release property has been known for some time, its functional importance to the integrated behavior of the cardiac ventricular myocyte has not been fully appreciated. We previously formulated a biophysically based model, in which LCCs and RyRs interact via a coarse-grained representation of the dyadic space. The model captures key features of local control using a low-dimensional system of ordinary differential equations. Voltage-dependent gain and graded Ca^2+^ release are emergent properties of this model by virtue of the fact that model formulation is closely based on the sub-cellular basis of local control. In this current work, we have incorporated this graded release model into a prior model of guinea pig ventricular myocyte electrophysiology, metabolism, and isometric force production. The resulting integrative model predicts the experimentally observed causal relationship between action potential (AP) shape and timing of Ca^2+^ and force transients, a relationship that is not explained by models lacking the graded release property. Model results suggest that even relatively subtle changes in AP morphology that may result, for example, from remodeling of membrane transporter expression in disease or spatial variation in cell properties, may have major impact on the temporal waveform of Ca^2+^ transients, thus influencing tissue level electromechanical function.

## Introduction

Since publication of the first computational model of the cardiac myocyte action potential (AP) in 1960 (Noble, 1960), the range of biological processes described in models of the cardiac myocyte has grown continuously. While the integrative nature of today’s most commonly used models differ, the sub-cellular processes for which there are quantitative, experimentally based models include: (a) voltage-gated ion channels and currents; (b) intracellular calcium (Ca^2+^) dynamics and Ca^2+^-induced Ca^2+^-release (CICR); (c) electrogenic and ATP-dependent membrane transporters; (d) regulation of intracellular Ca^2+^, sodium (Na^+^), potassium (K^+^), and hydrogen ion (H^+^) concentrations; (e) mitochondrial ATP production and its regulation; (f) coupling of ATP production to energy requiring membrane transporters and myofilaments; and (g) ligand gated membrane receptors and intracellular signaling pathways. However few models combine electrophysiology with contraction mechanics, mitochondrial energetics, or intracellular signaling due to the computational difficulty of combining disparate time and/or spatial scales. While the incorporation of more cellular component models increases the descriptive power of the combined model, the complexity of whole-cell model behavior and computational cost increase almost exponentially with the number of constitutive mechanisms represented.

The close interplay between modeling and experiments has enabled a remarkably deep understanding of the function of cardiac myocytes. In some cases, myocyte models not only reconstruct the experimental data on which they are based, they predict new emergent behaviors that have been validated subsequently by experiments. As a result, models now play a central role in understanding the relationships between molecular function and the integrated behavior of the cardiac myocyte in health and disease.

**Figure 1:**
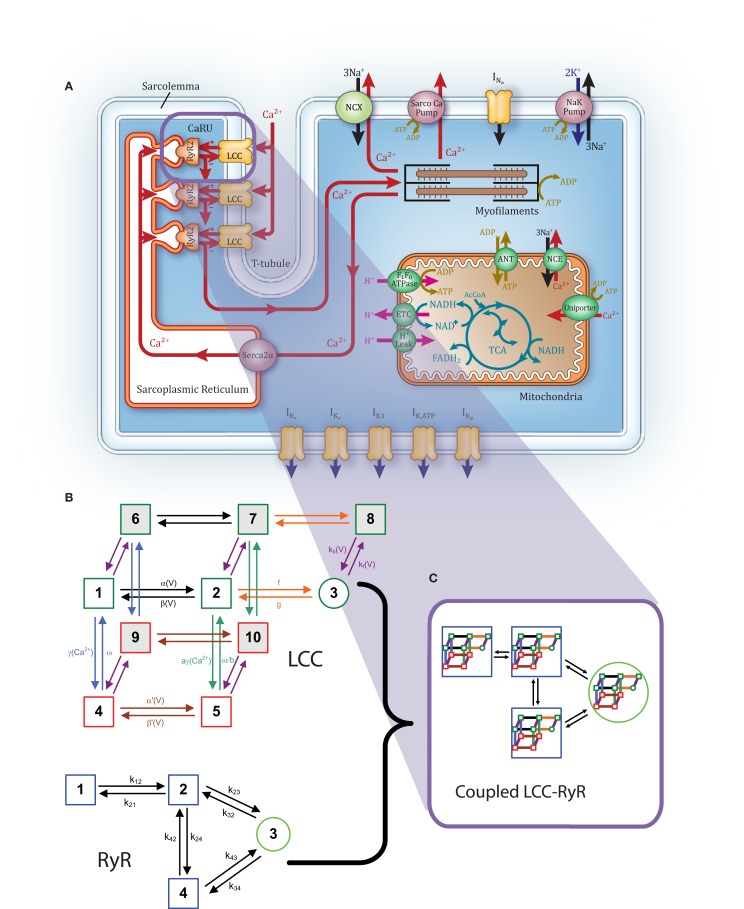
**Overview of guinea pig coupled model**. **(A)** Schematic illustration of the model structure. **(B)** LCC (top) and RyR (bottom) Markov model structures. **(C)** The 40-state LCC:RyR model representing each possible pairing of LCC and RyR states.

The most fundamental property of cardiac myocytes is that they are electrically excitable cells (they generate APs). The rapid increase in membrane depolarization during the early phase of the AP increases the open probability of sarcolemmal L-Type Ca^2+^ channels (LCCs). LCCs are preferentially located in the t-tubules (see Figure 1A), which are invaginations of the sarcolemma extending deep into the cell. Further, LCCs within the t-tubules are preferentially localized in structures known as dyads. Dyads are regions where t-tubule membrane is in close opposition to the sarcoplasmic reticulum (SR) membrane. The SR is a luminal organelle located throughout the interior of the cell. It is involved in uptake, sequestration, and release of Ca^2+^ in a process known as intracellular Ca^2+^ cycling. The junctional SR (JSR) is the portion of the SR most closely approximating the t-tubules. Ryanodine receptors (RyRs) are channels located in the JSR membrane in close opposition to LCCs in the dyad. During the initial phase of the AP, sarcolemmal membrane depolarization increases LCC open probability. The resulting flux of Ca^2+^ into the dyadic space (trigger flux) leads to Ca^2+^ binding to the RyRs. This increases RyR open probability, and when open, the resulting Ca^2+^ flux through RyRs (release flux) is directed into the dyadic space. There are thousands of dyads within the cardiac myocyte, and the net flux of Ca^2+^ from dyads into the cytosol triggers muscle contraction in a process known as excitation-contraction coupling (ECC). The ratio of release to trigger flux is typically large, and is referred to as ECC gain.

Graded release refers to the phenomenon, originally observed by Fabiato (1985), whereby Ca^2+^ release from JSR is a graded, smooth, continuous function of the amount of trigger Ca^2+^ entering the cell via LCCs. The majority of cardiac myocyte models lump all dyadic spaces together into a common pool known as the subspace, and net trigger flux through LCCs and net release flux through RyRs is directed into this common pool. In a landmark 1992 paper (Stern, 1992), Stern showed that the strong positive feedback effect on RyR open probability due to the fact that release flux is directed into the same pool of Ca^2+^ that serves as the trigger for RyRs (i.e., the common pool) results in all-or-none rather than graded release. More specifically, he showed that common pool models cannot reproduce both high gain and graded release. Despite this fact, early common pool models were able to reproduce a broad range of cardiac myocyte behaviors. However, Greenstein and Winslow (2002) showed that incorporation of new experimental data, demonstrating that Ca^2+^-dependent inactivation (CDI) of LCCs is stronger than voltage-dependent inactivation (VDI), into common pool models de-stabilized repolarization of the AP due to abnormal Ca^2+^ handling (here, de-stabilization means that at normal physiological pacing rates, APs could exhibit an oscillatory plateau phase, and large, irregular variation in AP duration, APD). Thus, common pool models not only fail to capture the graded release property, they cannot capture one of the most fundamental properties of normal cardiac myocytes at physiological pacing rates – stable APs.

Stern (1992) showed that graded release is achieved when it is assumed that LCCs can only trigger Ca^2+^ release from their adjacent RyRs in the dyad. Under this assumption, graded release arises as the result of statistical recruitment of release clusters, a process known as local control of Ca^2+^ release. Guided by this insight, Greenstein and Winslow (2002) showed that when local control is incorporated into ventricular myocyte models by simulating the stochastic gating of LCCs and RyRs in each dyad, AP properties are stabilized. However, one drawback of models based on systems of stochastic ordinary differential equations is that solution of these equations is computationally demanding. Hinch et al. (2004) resolved this problem by using the fact that the time rate of change of dyadic Ca^2+^ concentration is so fast relative to the time evolution of any other biological process in the models that it can be assumed to immediately reach its steady-state value. This simple, reasonable assumption enabled the graded release property to be modeled using a low-dimensional system of ordinary differential equations in which LCCs and RyRs behave as a strongly coupled system. Incorporation of this “coupled LCC-RyR model” into cardiac ventricular myocyte models enabled these models to achieve graded release with high gain and stable APs (Greenstein et al., 2006) in a more computationally efficient manner. The advantage of such models, as compared to models with phenomenological formulations of the release mechanism, is that they can be used to study the functional consequences of altered molecular function on ECC gain since this property emerges as a result of capturing fundamental biological detail. This is not true of phenomenological models formulated using ECC gain functions that are explicitly built into the models.

In 2003, Cortassa et al. (2003) formulated a computational model of cardiac mitochondria including descriptions of the tri-carboxylic acid (TCA) cycle and its regulation by Ca^2+^, oxidative phosphorylation, the F_1_-F_0_ ATPase, the adenine nucleotide translocator, the Ca^2+^ uniporter, the Na^+^-Ca^2+^ exchanger, and mitochondrial Ca^2+^ dynamics. In 2006, this model was integrated into a version of the Jafri–Rice–Winslow model of the guinea pig ventricular myocyte (Jafri et al., 1998) that had been extended to include a description of isometric force generation (Rice et al., 2000). This integrative ECC/mitochondrial energetics (ECME) model (Cortassa et al., 2006) also described coupling between mitochondrial ATP production and energy requiring membrane transporters, as well as control of mitochondrial energetics by cytosolic Ca^2+^. This model was able to reconstruct steady-state relationships between force generation and oxygen consumption at different stimulus frequencies, as well as rapid temporal changes in mitochondrial NADH and Ca^2+^ in response to abrupt changes in workload. Nonetheless, this model is a common pool model exhibiting non-physiological all-or-none Ca^2+^ release. Incorporating the graded release property into this model is important because mitochondria are bounded at each end by the JSR Ca^2+^ release sites, a close association that is supported by the observation that there are electron dense structures linking the mitochondrial outer membrane to t-tubules (Hayashi et al., 2009). This possible colocalization of mitochondria and the Ca^2+^ release sites implies that mitochondria may sense the local dyadic Ca^2+^ signal rather than the bulk cytosolic Ca^2+^ signal exclusively. In addition, mitochondria are “buffers” of Ca^2+^ by virtue of the presence of the Ca^2+^ uniporter in the inner mitochondrial membrane. Therefore, mitochondria may not only sense and be regulated by the large, fast, local dyadic Ca^2+^ signal, they may also act to buffer this signal, thereby influencing ECC (Maack et al., 2006).

As a first step toward investigating these important questions, we present an extension to the ECME model incorporating the coupled LCC-RyR formulation of graded release and description of the local Ca^2+^ signal. We demonstrate that this model of the guinea pig ventricular myocyte is able to reconstruct a broad range of experimental data. The model predicts that interactions between voltage-dependent properties of ECC gain and AP shape during the plateau phase have an important role in the timing of the Ca^2+^ transient and thus force generation. This prediction, which emerges from the underlying graded release model, is validated by experimental data. Further, we show that factors influencing AP plateau shape such as magnitude of the fast transient outward K^+^ current (in species other than guinea pig) can significantly affect timing of Ca^2+^ release. This model prediction is also validated by experimental data. These behaviors are specific to the graded release model, and cannot be revealed when using common pool models with all-or-none release. Finally, we show preliminary results indicating that the model predicts experimentally measured effects of mitochondrial Ca^2+^ uniporter block on amplitude of the cytosolic and mitochondrial Ca^2+^ transients, demonstrating the important role of beat-to-beat Ca^2+^ buffering by mitochondria.

## Materials and Methods

### The coupled LCC-RyR Ca^2+^ release unit

We have incorporated a coupled LCC-RyR model of CICR into the ECME guinea pig myocyte model of Cortassa et al. (2006; Figure 1A). The coupled LCC-RyR model of the Ca^2+^ release unit (CaRU) is based on that presented previously for canine myocytes by Greenstein et al. (2006; see Figures 1B,C). The CaRU is represented by a single LCC in the t-tubule membrane, a single RyR located in the closely opposed JSR membrane, and a dyadic volume in the space between them, which functions as a separate Ca^2+^ compartment (Figure 1A). The rate of Ca^2+^ diffusion from the dyadic space to the cytosol is sufficiently rapid allowing for the assumption that subspace Ca^2+^ levels equilibrate instantaneously and can therefore be expressed algebraically in terms of the fluxes through the LCC and RyR. Another simplification arises from the assumption that refilling of the JSR occurs quickly enough that the Ca^2+^ levels in the JSR can be assumed to be approximately equal to network SR (NSR) Ca^2+^ levels. In this minimal model, the single model RyR represents the estimated number of release channels per LCC measured in guinea pig (Bers and Stiffel, 1993), and thus corresponds to a cluster of five simultaneously gating RyRs. Therefore, unitary flux is increased to five times that of a single-channel. The CaRU model is made up of 40 states (Figure 1C), which represent all possible pairings of the 10 state LCC model and the four state RyR model. Further details on the coupled LCC-RyR formulation may be found in Greenstein et al. (2006).

**Figure 2:**
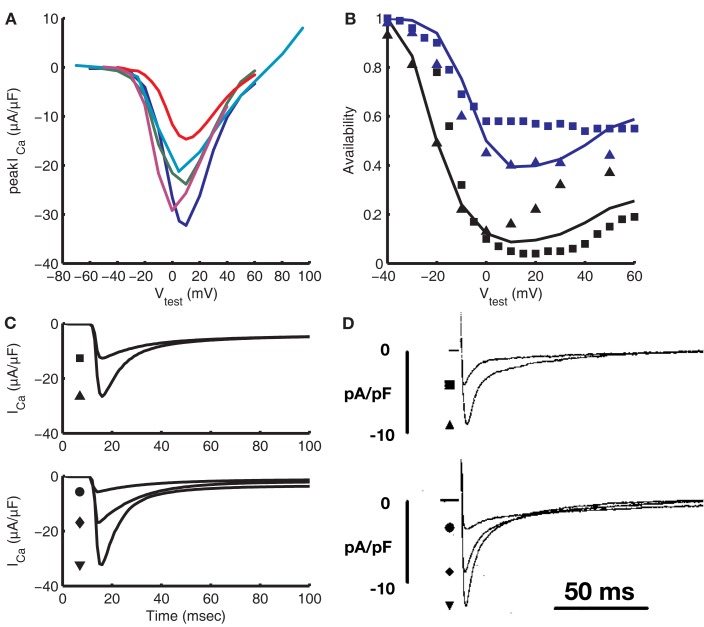
**Validation of the L-type Ca^2+^ current**. **(A)** Current-voltage relation for the model (blue) compared with experimental data from Rose et al. (1992; green), Linz and Meyer (1998; red), Grantham and Cannell (1996; teal), and Allen (1996; purple). Recordings from Rose et al. (1992), Linz and Meyer (1998), and Allen (1996) were adjusted to 37°C using the *Q*_10_ value from Cavalié et al. (1985). **(B)** Steady-state availability in the presence of CDI and VDI (black) and with VDI only (blue) from the model (lines) is compared to experimental data from Linz and Meyer (1998; squares) and Hadley and Lederer (1991; triangles). **(C)** Model traces of *I*_Ca,L_ vs. time for 400 ms test pulses from a pre-clamp of −40 mV. **(D)** Experimental *I*_Ca,L_ traces from Linz and Meyer (1998), also from a pre-clamp of −40 mV. For the top set of traces in **(C,D)**, the square represents a test potential of −10 mV and the triangle a test potential of 0 mV. For the bottom set, test potentials are 10, 30, and 50 mV for the circle, diamond, and triangle, respectively. **(D)** Was reproduced with copyright permission of John Wiley and Sons.

Figure 2 demonstrates kinetic and steady-state properties of the LCC model. Parameters of the LCC model were constrained using voltage-clamp data obtained from isolated guinea pig ventricular myocytes measured at 34–37°C. Figure 2A shows the L-type Ca^2+^ current peak *I*–*V* relationship. *I*_Ca,L_ is non-zero for test potentials between approximately −40 and +60 mV with a maximal peak of −32 μA/μF at +10 mV. The membrane potential at which the I–V curve peaks is in good agreement with data from four different guinea pig studies (Rose et al., 1992; Allen, 1996; Grantham and Cannell, 1996; Linz and Meyer, 1998). Peak current is −32 μA/μF at +10 mV, at the high end of measured values. For comparison, experiments show −21 μA/μF at 37°C (Grantham and Cannell, 1996), −25.68 μA/μF temperature-adjusted from 34 (Allen, 1996) to 37°C, and −24 μA/μF temperature-adjusted from 22 (Rose et al., 1992; Allen, 1996; Grantham and Cannell, 1996; Linz and Meyer, 1998) to 37°C, where adjustments are made using a *Q*_10_ value of 2.96 from Cavalié et al. (1985) Steady-state CDI (Figure 2B), was constrained using data from double-pulse voltage-clamp protocols (Hadley and Lederer, 1991; Linz and Meyer, 1998), with CDI being greater than VDI at all potentials. VDI properties were constrained using data from Linz and Meyer (1998), who measured a non-specific current through LCCs in a Ca^2+^-free solution, and from Hadley and Lederer (1991), who determined VDI from measurements of LCC gating current charge immobilization. Rate of recovery from VDI was constrained using double-pulse voltage-clamp data from isolated rabbit ventricular myocytes (Mahajan et al., 2008). Figure 2C shows the time course of *I*_Ca,L_ at various test potentials. The current peaks 3 ms after stimulus before decaying over approximately 100 ms to a value near zero. The time course of *I*_Ca,L_ recordings, including time to peak *I*_Ca,L_, are in good agreement with data from Linz and Meyer (1998; Figure 2D). Different peak magnitudes between model and experimental results suggest differing channel density, which is also reflected in Figure 2A. The number of LCCs (and thus release units) in the model was set to 339,000 in order to match experimental data on fractional release, as discussed in Section “CICR During the Action Potential” below. This number of LCCs is between the estimate of ≈276,000 predicted by binding experiments (Bers and Stiffel, 1993) and the estimate of ≈500,000 from LCC gating current studies (Hadley and Lederer, 1991).

**Figure 3:**
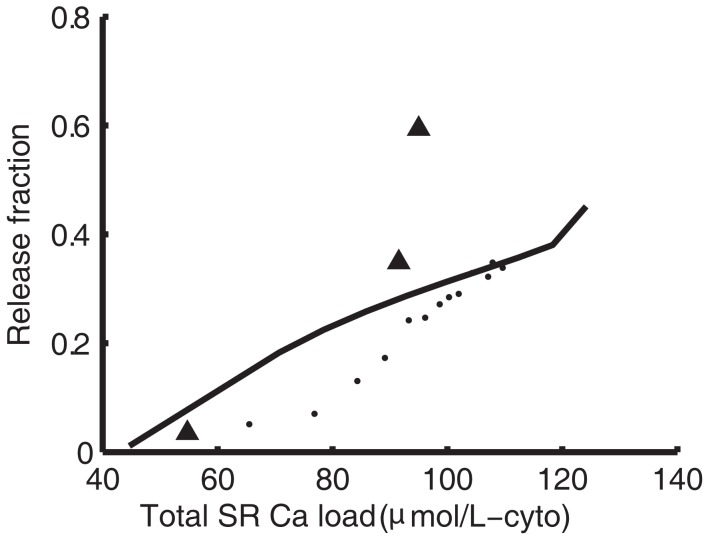
**Sarcoplasmic reticulum load-dependence of fractional release**. Model SR load vs. fractional release (solid line) compared to experimental data from Shannon et al. (2000) in rabbit (dots) and Bassani et al. (1995) in ferret (triangles).

Ryanodine receptor properties are those from our previous model (Greenstein et al., 2006). Briefly, the RyR model comes from a formulation by Rice et al. (1999), which was modified from a model by Keizer and Smith (1998; Figure 1B). Upon elevation of subspace Ca^2+^ levels, RyRs rapidly transition from state 1 through state 2 into state 3, the open state. Termination of release occurs as the channel transitions from state 3 to state 4, the inactive state, where it remains until subspace Ca^2+^ levels drop and the RyR returns to state 1. Within the context of the whole-cell model, this RyR model produces an increasing load-dependent fractional release relationship. Figure 3 shows the fraction of total SR Ca^2+^ released by an AP at varying SR loads. The model relationship is qualitatively similar to experimental data from rabbit (Shannon et al., 2000) and ferret (Bassani et al., 1995), two species which have strong Na^+^-Ca^2+^ exchanger (NCX) contribution to relaxation, resulting in Ca^2+^-cycling that is more similar to guinea pig than rat or mouse.

### Ion channels and Ca^2+^ cycling

**Figure 4:**
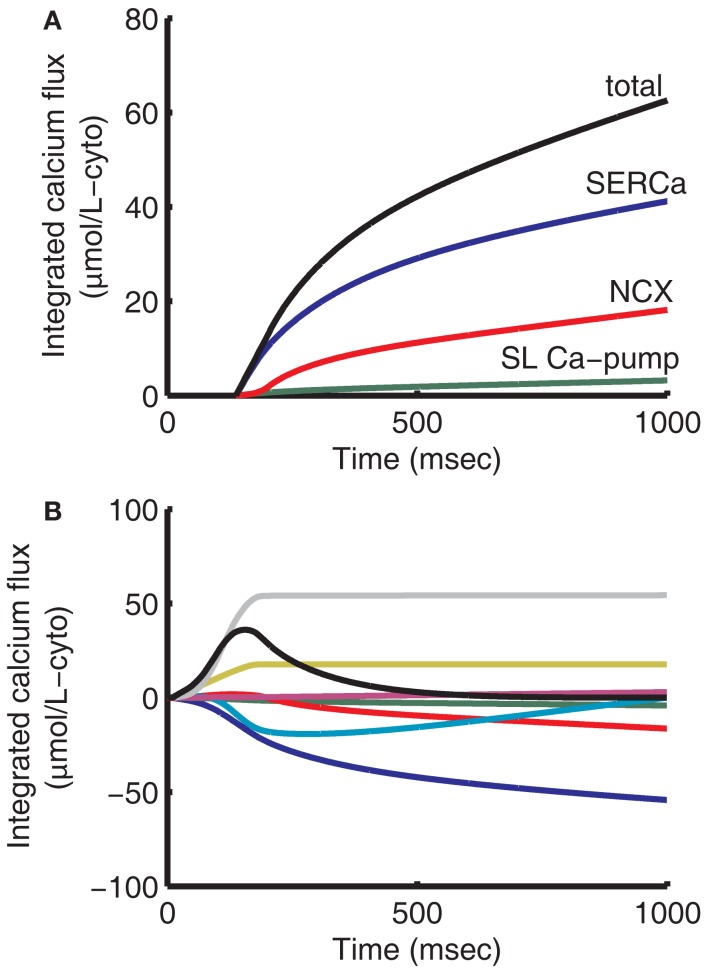
**Integrated cytosolic Ca^2+^ fluxes**. Cytosolic Ca^2+^ uptake is given as the sum of SERCa, NCX, and sarcolemmal Ca^2+^-pump fluxes **(A)**. SERCa contributes 65.9% of uptake, NCX 28.9%, and SL Ca-pump 5.1%. In this model mitochondria contribute significant beat-to-beat buffering. The mitochondrial uniporter predominates during the first 300 ms of the cycle, resulting in a net uptake of Ca^2+^ from the cytosol **(B)**. For the remainder of the cycle, mitochondrial Na^+^-Ca^2+^ exchanger predominates, resulting in Ca^2+^ being transferred back out of the mitochondria to the cytosol. The mitochondria release an amount of Ca^2+^ equal to 12.7% of the net integrated Ca^2+^ uptake flux into the cytosol during relaxation. Integrating over the entire 1000 ms period **(B)**, fluxes into the cytosol are given as positive and fluxes removing Ca^2+^ from the cytosol are negative. Fluxes shown represent SERCa (blue), NCX (red), SL Ca-pump (green), mitochondria (teal), background Ca^2+^ current (magenta), L-type Ca^2+^ current (yellow), SR release (gray), and total (black).

Models for the remaining (non-dyadic) channels, pumps, and exchangers are based on those of the 2006 ECME model (Cortassa et al., 2006) with the following changes. The previous formulation of the delayed rectifier potassium current *I*_K_ is replaced with the formulation of Zeng et al. (1995) that separates this current into two components, the rapid and slow (*I*_Kr_ and *I*_Ks_) delayed outward rectifier currents. *I*_Kr_ and *I*_Ks_ current amplitudes were adjusted to match model APD to experimental data (see Results). The equation for *I*_Kp_ was modified to better fit experimental data (Yue and Marban, 1988). The NCX model was updated with the model of Weber et al. (2001), which incorporates allosteric regulation by cytosolic Ca^2+^. NCX affinity constants for allosteric Ca^2+^ regulation and for intracellular Na^+^ were modified slightly to fit guinea pig NCX voltage dependant behavior (Maack et al., 2005) and diastolic Ca^2+^ levels under voltage-clamp (Han et al., 1994; Isenberg and Han, 1994). This allowed the model to maintain appropriate diastolic Ca^2+^ levels while still achieving a time constant of SR load rest decay of approximately 80 s (not shown), in qualitative agreement when compared with experimental data exhibiting a tau of 36 s (Terracciano et al., 1995). A mitochondrial Na^+^-H^+^ exchanger (Wei et al., 2011) was also added, which helps to ensure conservation of Na^+^ between the cytosolic and mitochondrial compartments. Ca^2+^ cycling was modified by adjusting NCX magnitude to achieve the approximately 60:40 balance of Ca^2+^ resequestration vs. Ca^2+^ export fluxes during diastole at a pacing frequency of 1 Hz (Bers, 2001). The ATP-dependent K^+^ current (*I*_K(ATP)_) was also added using a model by Ferrero et al. (1996) to incorporate the effects of ATP on APD. The conductance of *I*_K(ATP)_ was modified to take into account the range of experimental data (Nichols and Lederer, 1990; Weiss et al., 1992). Minor changes were made to rate-limiting mitochondrial parameters in the TCA cycle, respiratory chain, and adenine nucleotide transporter to increase ATP supply at higher pacing frequencies. Ca^2+^-regulation of the mitochondria remains unchanged. Ca^2+^ uptake fluxes during 1 Hz pacing were numerically integrated at 1 ms resolution from the peak of the Ca^2+^ transient to the end of diastole (see Figure 4A) to simulate experimental relaxation protocols (Bers, 2001). Comparing the total moles of Ca^2+^ removed from the cytosol, the SR Ca^2+^-ATPase (SERCa) takes up 65.9% of the transported cytosolic Ca^2+^, NCX removes 28.9%, and the sarcolemmal (SL) Ca-pump removes 5.1%, close to estimates of 67% SERCa, 30% NCX, and 3% slow processes (Bers, 2001). The mitochondrial uniporter removes Ca^2+^ from the cytosol most effectively during the upstroke of the Ca^2+^ transient, while Ca^2+^ extrusion by the mitochondrial Na^+^/Ca^2+^ exchanger dominates during relaxation (see Figure 4B). The result is that a bolus of Ca^2+^ 12.7% of the size of the net integrated Ca^2+^ flux is directed back into the cytosol during relaxation. This illustrates the buffering role that mitochondria in this model play during Ca^2+^ cycling. Recirculation fraction, calculated as the amount of Ca^2+^ contributing to the Ca^2+^ transient that is derived from the SR stores at steady-state, is 69%. This is close to estimates between 63 and 67% (Bers, 2001). At steady-state, the amount of Ca^2+^ released from the SR must equal the amount resequestered during a given beat (see Figure 4B). Analogously the amount of Ca^2+^ entering the cell across the cell membrane must be extruded during relaxation. Thus the ratio of cytosolic Ca^2+^ extruded during relaxation to the amount resequestered into the SR is a good approximation of the ratio of Ca^2+^ flux into the cell across the sarcolemma to SR Ca^2+^ release. Here we have approximately 31% of cytosolic Ca^2+^ due to transsarcolemmal influx and 69% due to SR release. As expected at steady-state, total and mitochondrial integrated fluxes start and end at zero (see Figure 4B). Due to conservation of Ca^2+^, at steady-state the integrals of inbound and outbound fluxes across the sarcolemma also sum to zero.

### Computational methods

Model code was written in C++ using the SUNDIALS CVODE integration library and run using an IBM PC workstation with a 2.80-GHz processor and 2.5 GB of RAM. On such a workstation, this 75-state model takes approximately 15 s to compute 10 1 Hz APs with no output written to files. This is comparable to the performance of current models for which code is available (Shannon et al., 2004; Faber et al., 2007). Code and additional information are available online (http://www.icm.jhu.edu/models/). Equations and parameters for the model are also available in the online supplement.

## Results

### CICR during the action potential

**Figure 5:**
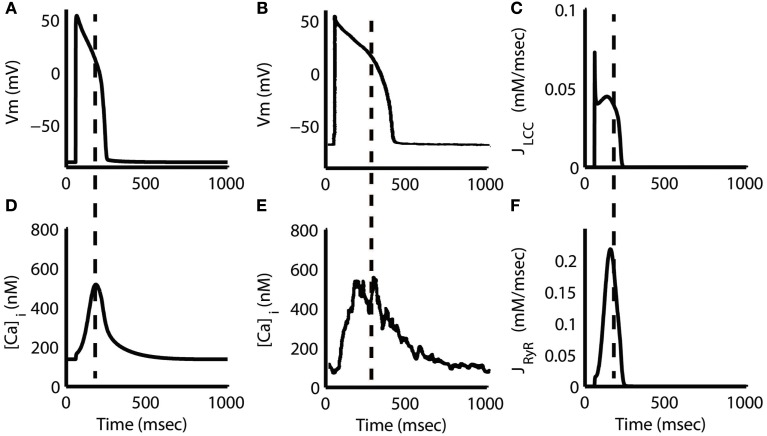
**Action potential and Ca^2+^ transient**. Steady-state AP at 1 Hz pacing from the model **(A)** and experiment (Sipido et al., 1995b) **(B)** along with the corresponding Ca^2+^ transients **(D,E)**. Note that the peak of the model transient is aligned with the middle of the AP plateau phase (dashed line), approximately 127 ms after stimulus. Experimental data in **(E)** show a similar delay in the Ca^2+^ transient peak of approximately 190 ms. L-type Ca^2+^ flux (J_LCC_) **(C)** shows a large, but brief peak aligned with the initial AP depolarization followed by a slow peak during the AP plateau. RyR flux **(F)** increases slowly and reaches its maximum in parallel with the J_LCC_ slow peak and AP plateau. Dashed lines in **(C,F)** correspond to the time of Ca^2+^ transient peak from **(D)**. Flux measurements are given with respect to subspace volume. **(B,E)** Were reproduced with copyright permission from The Physiological Society.

Figure 5A shows the model AP, which has a steady-state duration of 189 ms at 1 Hz pacing. This is consistent with experimental recordings obtained near the physiological temperature of 37°C with a 1-Hz APD range of approximately 130–180 ms (Arreola et al., 1991; Sicouri et al., 1996; Chen et al., 2000). AP shape, including resting membrane potential, peak voltage, and phase 2 and 3 AP slope, are in good correspondence with these experimental data.

The model Ca^2+^ transient begins to increase as soon as *I*_Ca,L_ is triggered, but the peak is delayed 119 ms with respect to the peak voltage of the AP (Figures 5A,D). A delay of 144–190 ms is supported by experimental recordings (Sipido et al., 1995b; Grantham and Cannell, 1996; see also Figures 5B,E) and by the prediction of local control theory that local fidelity of ECC decreases at highly depolarized potentials, such as those that occur early in the guinea pig AP. At highly depolarized potentials, the driving force for Ca^2+^ current through LCCs is reduced, even though whole-cell LCC flux may be large (Figure 5C). The resulting smaller unitary currents are less likely to trigger release via RyRs, and hence ECC gain is reduced. This counteracts the increase in gain resulting from increased LCC open probability at more depolarized potentials. The model predicts that triggering of SR Ca^2+^ release is lowest during the early phases of the AP, and increases slowly as the AP evolves (Figure 5F) because of this initial low gain. This leads to a relative delay in the peak of the Ca^2+^ transient waveform as compared to other species. In contrast, the Greenstein et al. (2006) canine model, which also features the coupled LCC-RyR formulation of CICR, exhibits a cytosolic Ca^2+^ transient that peaks approximately 80 ms earlier. The difference can be attributed to the presence of an early repolarization phase in the canine AP, which quickly hyperpolarizes the membrane potential into the range where ECC gain is maximal. The guinea pig models of Faber et al. (2007) and Gaur and Rudy (2011), which represent the same species as the present model, but use different release formulations, also demonstrate earlier cytosolic Ca^2+^ peaks than shown in this model (see Discussion for a full analysis).

**Figure 6:**
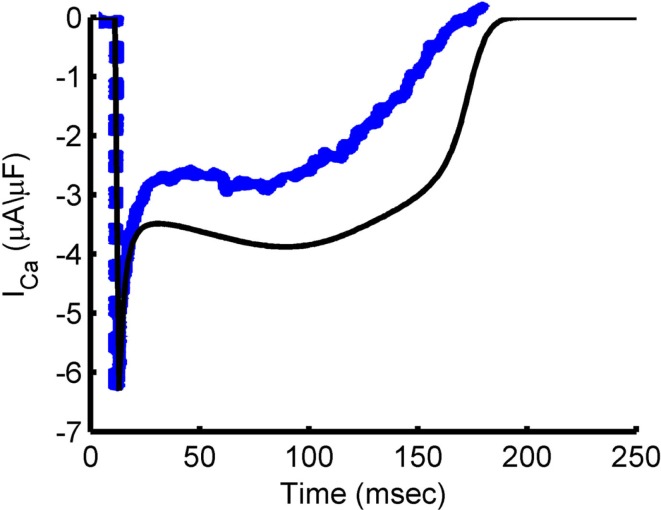
***I*_Ca,L_ during the AP**. Simulated *I*_Ca,L_ trace during steady-state AP at 1 Hz pacing (black) and experimental *I*_Ca,L_ trace from a guinea pig ventricular myocyte undergoing 1 Hz pacing at 35–37°C (blue). Blue trace is reproduced from Grantham and Cannell (1996) by copyright permission of the American Heart Association.

**Figure 7:**
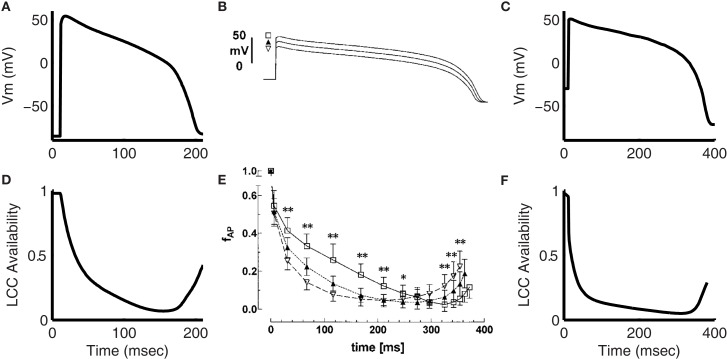
**Inactivation of the L-type Ca^2+^ current during the AP**. **(A)** Model 1 Hz AP. **(D)** Simulated LCC availability during the AP shown in **(A)**. Experimental data from Linz and Meyer (1998) showing the AP clamp waveform **(B)** and LCC availability during the AP clamp **(E)** for guinea pig myocytes at 35°C. **(C)** Shows model membrane potential output using the trace from **(B)** as an AP clamp. The LCC availability during the AP clamp is given in **(F)**. **(B,E)** Were reproduced by copyright permission of John Wiley and Sons.

Figure 6 shows the characteristic fast peak, early decay, and late peak shape of the guinea pig model *I*_Ca,L_, which is in good correspondence with experimental recordings (Arreola et al., 1991; Grantham and Cannell, 1996). Following the early decay, the current magnitude recovers partially in response to the increased driving force as the AP voltage drops, but the extent of this recovery is limited by increased Ca^2+^ release from the SR causing CDI. As the membrane potential repolarizes, the L-type current undergoes both VDI, as well as CDI resulting from SR Ca^2+^ release (see Figure 2B). LCC availability during the AP is shown in Figure 7. This quantity represents the occupancy of non-inactivated states in the channel model. The relation of LCC availability calculated from the model to membrane potential follows closely that of Linz and Meyer (1998). The kinetics vary between model and experiment due to the long APD in the experimental data. The APD_90_ from the experimental data is 380 ms, which more closely resembles the APD of a midmyocardial cell (Szigligeti et al., 1996), while the model is more representative of an epicardial cell. Simulations were also performed using the AP recording from the Linz and Meyer (1998) data as a voltage-clamp (Figures 7C,F). These simulations also show a rapid loss of availability, decreasing to a minimum of approximately 5% during the AP and a slow recovery of availability during the end of the repolarization phase.

**Figure 8:**
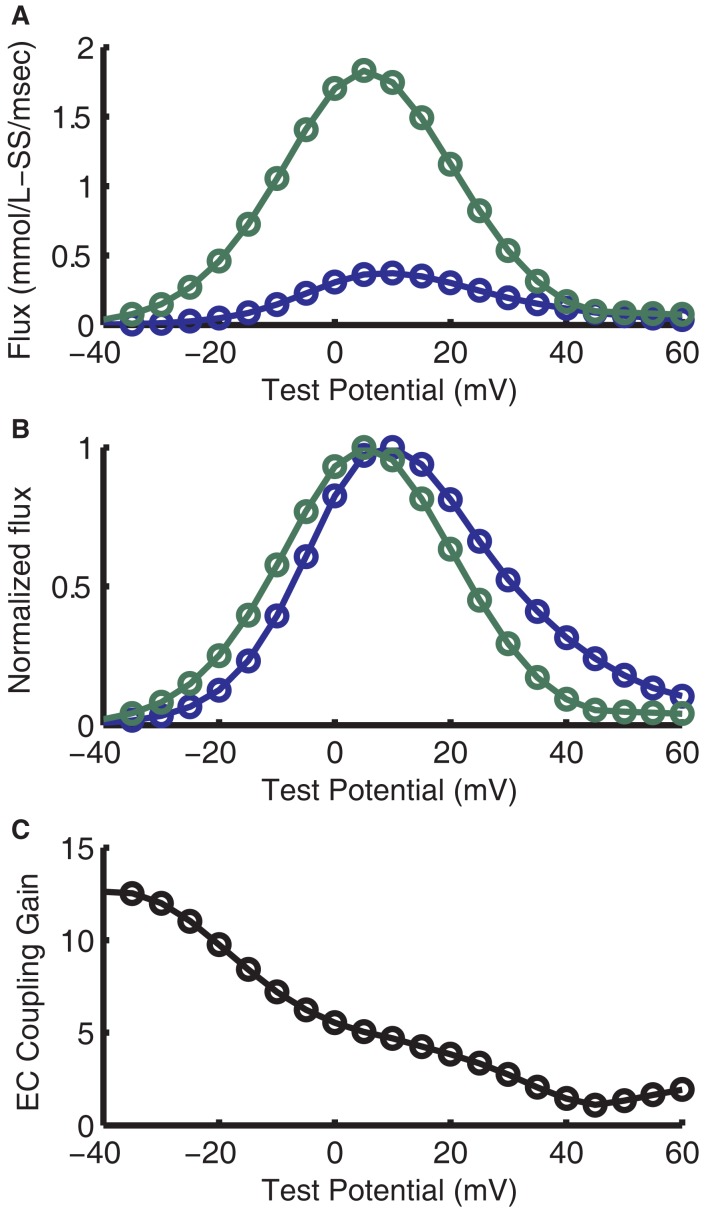
**Voltage-dependence of flux through LCCs and RyRs and ECC gain**. **(A)** Voltage-dependence of maximal Ca^2+^ flux through LCCs (blue) and RyRs (green). **(B)** Normalized fluxes from **(A)**. **(C)** ECC gain, as formulated by the ratio of maximal LCC flux to maximal RyR flux.

The purpose of introducing the local control CaRU model is to incorporate a biophysically realistic representation of graded release in the myocyte model. As shown in Figure 8A, the RyR release and LCC trigger fluxes are smooth, continuous functions of membrane potential. The RyR release flux exhibits a peak that is displaced in the hyperpolarizing direction from that of the LCC trigger flux, a key feature of experimental measurements of Ca^2+^ release. This is again seen in the normalized RyR release flux (Figure 8B), better illustrating the representative leftward shift with relation to the LCC flux, as seen in the experimental data from Wier et al. (1994). Here, the LCC flux peaks at +10 mV while the RyR flux peaks at +5 mV. It is this shift that results in the characteristic monotonically decreasing gain function (Figure 8C). Voltage-dependent gain has not been measured in guinea pig, but the gain at 0 mV is close to experimental estimates in guinea pig (Sipido and Wier, 1991; Rocchetti et al., 2005).

At 1 Hz pacing, the fractional release of total Ca^2+^ from the SR is approximately 33% (not shown). This quantity was calculated as unity minus the ratio of total systolic SR Ca^2+^ to total diastolic SR Ca^2+^. The 33% measurement given by the model agrees with 35% estimated in ferret (Bassani et al., 1995), a species with a similar recirculation fractional as the guinea pig (Bers, 2001). The peak RyR open probability is 2.9% at 1 Hz. As described by Bers (2001), given an experimentally measured peak release flux of 3 mM/s (Wier et al., 1994) and a unitary RyR flux near 0.4 pA (Mejía-Alvarez et al., 1999), only 40,000 RyRs need to be open at the time of maximal release. This is only 2% of the number of total RyRs calculated by Bers and Stiffel (1993).

**Figure 9:**
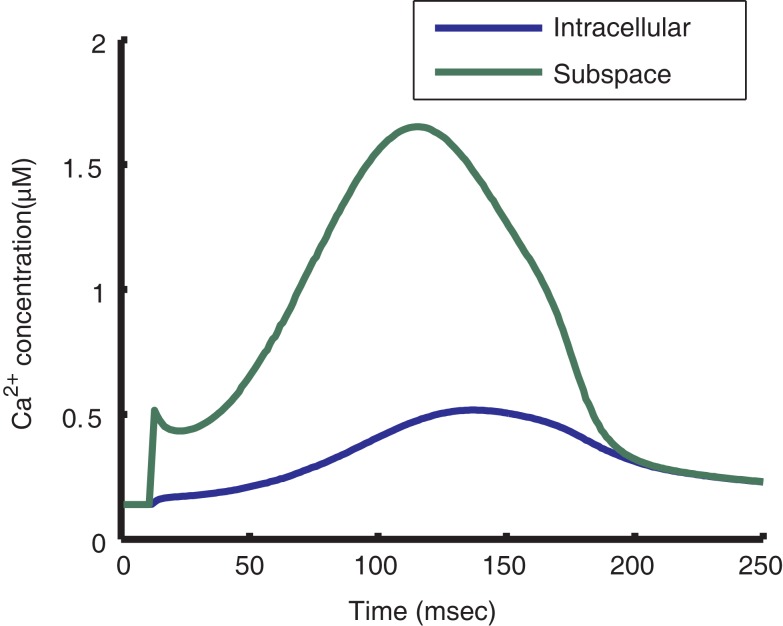
**Predicted subspace Ca^2+^ levels**. Model cytosolic Ca^2+^ transient during a steady-state 1 Hz AP (blue) and subspace Ca^2+^ transient averaged across all dyads (green). While the average subspace Ca^2+^ is approximately four times higher than that of the cytosol, the maximum subspace Ca^2+^ for a single dyad may reach 45 μM, measured as the maximum subspace Ca^2+^ for the open-open LCC-RyR configuration during a release event.

Incorporation of the local control model of the CaRU into the myocyte model allows for the prediction of localized subspace Ca^2+^ levels (Figure 9). The model calculates subspace Ca^2+^ for four different dyad macrostates: with LCC and RyRs closed, with only the LCC open, with only the RyRs open, and with both the LCC and RyRs open. Average subspace Ca^2+^ can be estimated by summing over the predicted Ca^2+^ concentrations for these four scenarios, weighted by their respective probability of occurrence. During 1 Hz pacing, the predicted average subspace Ca^2+^ level peaks near 2 μM, four times higher than the peak of the cytosolic transient. Subspace Ca^2+^ for dyads with open LCCs and RyRs reaches a maximum of 45 μM during the AP plateau.

### APD restitution

Action potential duration restitution describes the electrical response of the myocyte to a premature stimulus. When a myocyte is paced to steady-state at a constant basic cycle length (BCL), APD becomes constant from beat to beat. The time between the end of the AP and the onset of the next stimulus is the diastolic interval (DI). Immediately following an AP the cell will be in an inexcitable refractory state. As the DI increases beyond this refractory period toward the steady-state DI, an AP can be triggered, but its duration is less than that of the steady-state AP due to incomplete recovery from inactivation of *I*_Ca,L_ and *I*_Na_. At the tissue level, slow APD restitution and high pacing frequency can lead to the formation of a functional conduction block as the wave of depolarization catches up with refractory tissue from the previous beat. This block fails to excite and changes the direction of wavefront propagation. Additionally, the relationship between DI and APD has been shown to influence whether such aberrant conduction patterns damp out or devolve into arrhythmias (Qu et al., 1999; Garfinkel et al., 2000).

**Figure 10:**
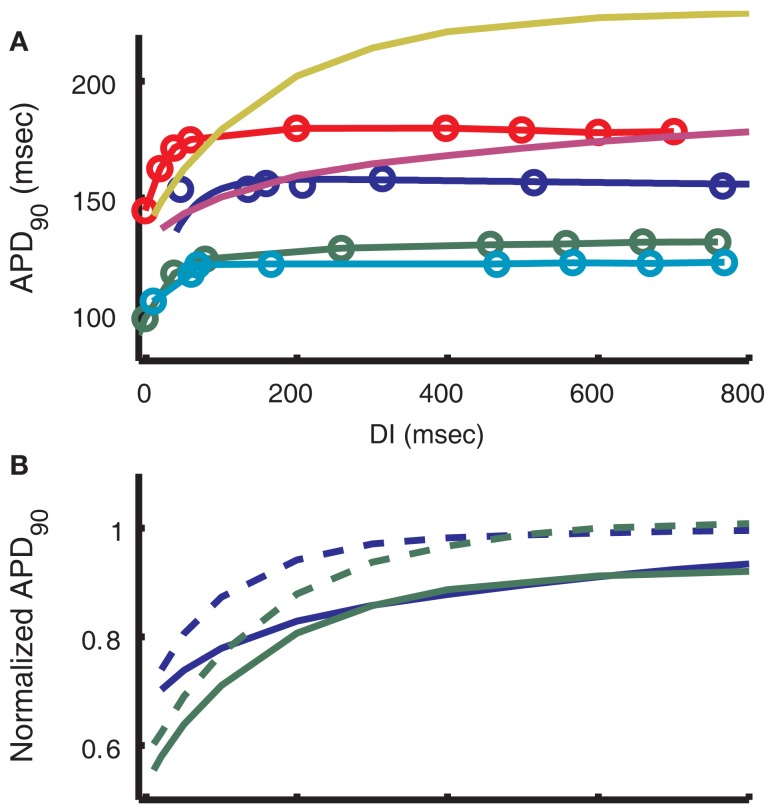
**Restitution of APD**. **(A)** APD restitution curves from guinea pig experimental data and models. Blue dots show 2000 ms BCL data from Bjornstad et al. (1993) connected by the biexponential fit reported by those authors. Green, red, and teal respectively show epicardial, midmyocardial, and endocardial experimental data from Sicouri et al. (1996). The purple line depicts output from the LRd07 model (Faber et al., 2007) and yellow is from the present model. A standard S1–S2 protocol was used to simulate APD restitution, starting from 0.5 Hz steady-state as in the protocol used by Sicouri et al. (1996). The single exponential time constant for restitution of the present model is 165 ms, compared with approximately 40 ms for Bjornstad et al. (1993) and approximately 46, 27, and 31 ms for Sicouri et al. (1996) epicardial, midmyocardial, and endocardial, respectively. **(B)** Alteration of the model restitution curve by reverting to the unmodified IKs formulation from Zeng et al. (1995). Blue is LRd07 model, green is the present model. Solid lines are simulations using the latest IKs formulation (one fast gate, one slow), dashed lines are for simulations with 1995 IKs formulation (two fast gates). Curves are normalized to the 2000-ms steady-state APD_90_ for each model, respectively.

An electrical restitution curve was generated for the model by pacing to steady-state at 2000 ms BCL and then saving the state of the model at the end of an AP. Using the state values from this time as initial conditions, premature stimuli at increasing DIs were applied and the resulting APD_90_ was measured. The results are compared to recordings under the same protocol from (midmyocardial) strips of guinea pig ventricle at 37°C (Figure 10A). A single exponential fit to the model results (yellow curve in Figure 10A) yields a time constant of 165 ms. This differs from measurements by Sicouri et al. (1996) and Davey et al. (2001) which are 26.7 and 78.4 ± 12.1 ms respectively for single guinea pig myocytes at 32°C. Possible causes of this discrepancy are presented in Section “Critique of the Model” below.

### Frequency-dependence of APD and ECC

To determine the frequency-dependence of APD and accumulated force, the pacing protocol of Szigligeti et al. (1996) was followed. Briefly, the model was first paced to steady-state at 3000 ms BCL. Pacing frequency was then increased in a stepwise manner. APD, peak cytosolic Ca^2+^ and accumulated force were recorded after 3 min at 3000, 2000, 1500, 1000, 700, 500, and 300 ms BCL.

**Figure 11:**
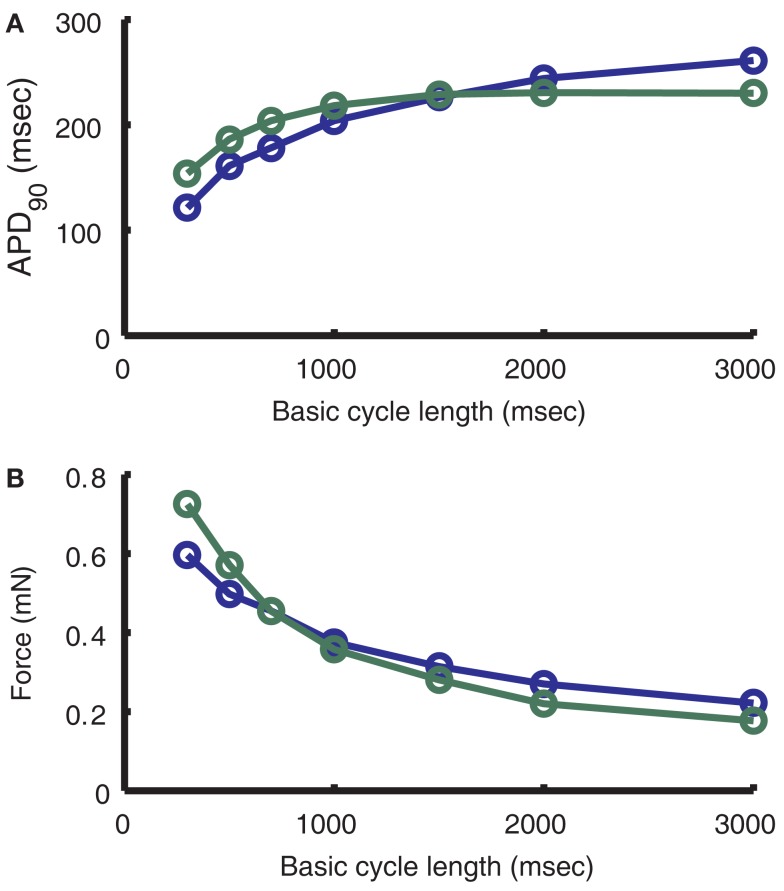
**Frequency-dependence of APD and Force**. **(A)** Comparison of model (blue) dependence of APD on frequency to that of Szigligeti et al. (1996; green). **(B)** Comparison of model (blue) dependence of force on frequency to that of Szigligeti et al. (1996; green). See Section “Frequency-Dependence of APD and ECC” of the text for conversion of normalized model output to force units.

The model results presented in Figure 11A (blue line) show a decrease in APD with increasing pacing frequency, from an APD_90_ of 261 ms at 3000 ms BCL to an APD_90_ of 114 ms at 300 ms BCL. The rate of decrease of APD with increasing frequency follows closely the data of Szigligeti et al. (1996; Figure 11A; green line). Experimental studies have shown that intracellular sodium ([Na^+^]_i_) varies as a function of pacing frequency (Cohen et al., 1982; Wang et al., 1988; Maier et al., 1997). The model exhibits similar frequency-dependence of Na^+^ levels with average [Na^+^]_i_ near 5.5 mM at 3000 ms BCL increasing to 8 mM by the end of the Szigligeti protocol. This Na^+^ accumulation is mediated through a frequency-dependent change in the balance of the Na^+^ entry via the fast sodium current (*I*_Na_) that initiates the AP and the export of Na^+^ by the Na^+^-K^+^ pump during diastole. In this model, NCX plays a large role in early repolarization of the plateau at slow frequencies. As [Na^+^]_i_ continues to decrease with an increase in BCL, outward NCX current is reduced, allowing prolongation of the AP plateau phase.

The force model used here is the same as that implemented previously in the ECME model (Cortassa et al., 2006). The frequency-dependence of peak force follows closely the data from Szigligeti et al. (1996) in guinea pig papillary muscle (Figure 11B). As BCL decreases, isometric force increases exponentially as a result of the underlying increase in peak cytosolic Ca^2+^ concentration associated with increased SR load and gain. To compare the normalized force output of the model with those data the cross-sectional area used is 0.013 mm^2^, which is equivalent to a fiber with an elliptical profile of width 0.24 mm and thickness 0.069 mm, the minimum of the range of measurements for the Cortassa et al. (2006) right ventricular trabecula samples. The frequency-dependence of both cytosolic Ca^2+^ and isometric force play an important part in the ability of the model to reproduce experimental results on energy supply during work transitions (see below). Both cytosolic Ca^2+^ and force transients show faster peaks at higher pacing frequencies, as has been shown experimentally (Layland and Kentish, 1999; Bluhm et al., 2000; Davey et al., 2001; Raman et al., 2006; not shown). Cytosolic Ca^2+^ transients also show frequency-dependent acceleration of relaxation (not shown), such that the time of decay to half peak amplitude is decreased with increasing frequency. Force transients have been shown to have a similar property (Bers, 2001; Davey et al., 2001), but the rate of force transient relaxation in the model does not vary significantly across pacing frequencies.

### Mitochondrial energetics

Control of mitochondrial energy production is mediated via the Ca^2+^ sensitivity of key enzymes in the tri-carboxylic acid (TCA) cycle, and through regulation of the F_1_-F_0_ ATPase by ADP. As pacing frequency increases, a higher ADP:ATP ratio results from the increased ATP consumption at rapid contraction rates. Increased mitochondrial ADP levels stimulate the F_1_-F_0_ ATPase to generate ATP by utilizing the proton-motive force as an energy source. Flux of electrons through the electron transport chain would in and of itself deplete the NADH pool. However, the amplitude of the cytosolic Ca^2+^ transient also increases with pacing frequency, and this Ca^2+^ signal is communicated to the mitochondria via Ca^2+^ uptake by the mitochondrial Ca^2+^ uniporter. Elevated mitochondrial Ca^2+^ levels stimulate the TCA cycle to increase production of NADH to sustain a higher rate of respiration. As in the original ECME model (Cortassa et al., 2006), the rate of respiration for the model presented here increases with increasing pacing frequency. Respiration rate increases 3.3× between 4000 ms BCL pacing and 300 ms pacing, similar to Cortassa et al. (2006; their Figure 4A).

During abrupt changes in pacing frequency, the mechanism described above results in NADH transients with complex kinetics. While increased mitochondrial Ca^2+^ levels stimulate NADH supply, this occurs with slower kinetics than the increase in demand. The result is an abrupt decrease in NADH before restoration to a new steady-state at the higher pacing frequency. For decreases in pacing frequency, ATP demand drops causing an overshoot in NADH levels, since production is still stimulated by high mitochondrial Ca^2+^. Cytosolic Ca^2+^ transient amplitudes drop and mitochondrial Ca^2+^ levels eventually follow as Ca^2+^ is pumped out of the mitochondria by the mitochondrial Na^+^-Ca^2+^ exchanger. After tens or hundreds of seconds NADH levels reach a steady-state corresponding to the slower pacing frequency.

**Figure 12:**
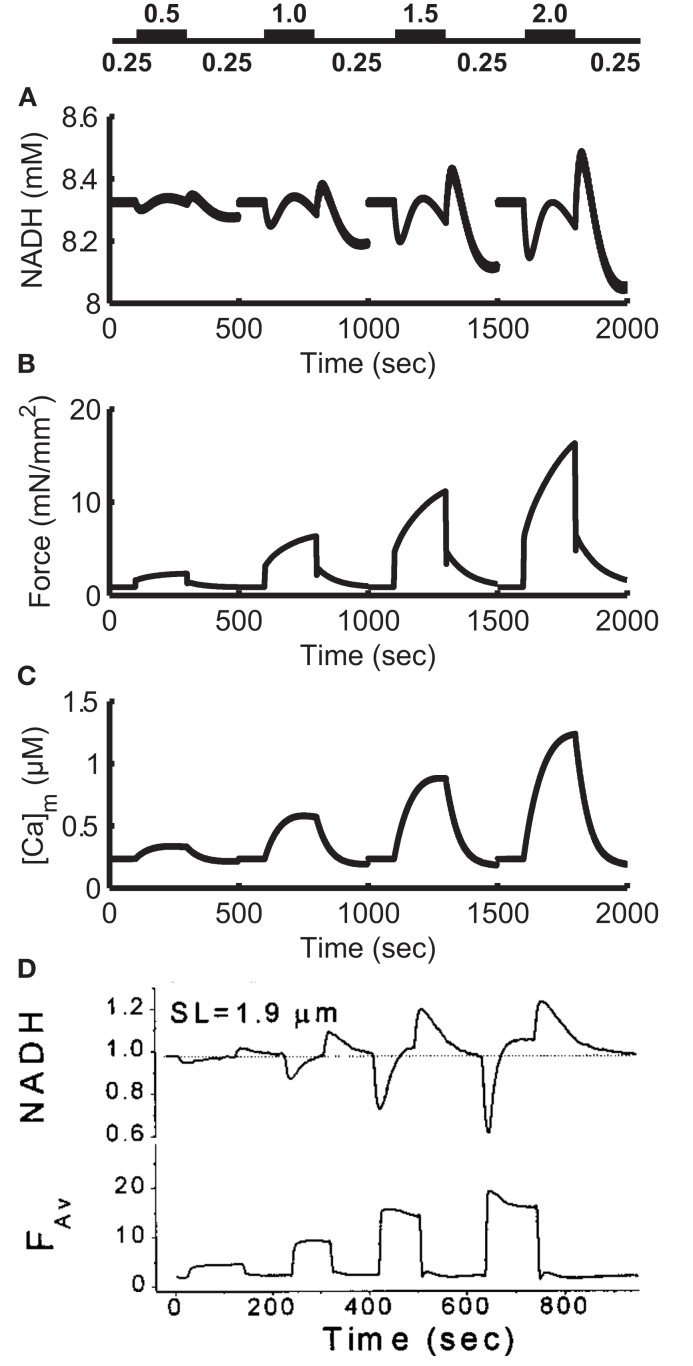
**Frequency-dependence of NADH levels**. **(A)** Simulation of NADH concentration for a pacing protocol consisting of 100 s at 0.25 Hz [see labels above **(A)**], 200 s at higher pacing frequencies of 0.5, 1.0, 1.5, and 2.0 Hz then 200 s recovery at 0.25 Hz. Each 500 s protocol is started from 0.25 Hz steady-state initial conditions. **(B)** Moving average of model force output using a 4000-ms window. Force ranges from approximately 0.9 mN/mm^2^ at 0.25 Hz to 16.4 mN/mm^2^ at the end of the 2-Hz pacing period. **(C)** Model mitochondrial Ca^2+^ concentration. **(D)** Experimental data from Brandes and Bers (1999) follow a similar protocol. NADH signal is normalized to 0.25 Hz level. **(D)** Was reproduced from Brandes and Bers (1999) by copyright permission of the Biophysical Society.

Figures 12A–C shows the response of the model to changes in workload. To simulate changes in heart rate, the model is paced at 0.25 Hz for 100 s, then pacing frequency is increased to a high workload frequency for 200 s before being allowed to recover at 0.25 Hz for another 200 s. This protocol is repeated for high workload frequencies of 0.5, 1, 1.5, and 2 Hz. Simulated NADH levels (Figure 12A) show an undershoot upon initiation of the high frequency stimulation, followed by a recovery to higher levels. Upon return to 0.25 Hz pacing, NADH levels exhibit an overshoot before beginning recovery to the steady-state 0.25 Hz level. In the model, NADH recovery to a steady-state is slow, taking approximately 600 s. The simulation results in Figure 12A were performed using the same experimental protocol. As a result, model NADH levels do not reach a steady-state within 200 s. While the kinetics and waveform of the NADH transients in the model are qualitatively similar to experimental data (Figure 12D), the lack of quantitative correspondence between the magnitudes of under- and over-shoots in our model, and the data of Brandes and Bers (1999), also seen in the original ECME model (Cortassa et al., 2006), may arise from the effects of ADP compartmentation. The smaller transient effects seen in the model indicate that the changes in workload in the cytosol are not inducing sufficiently large changes in the mitochondrial ADP pool. Averaged force (Figure 12B) shows an increase during high workload frequencies, up to 16.4 mN/mm^2^ at the end of 2 Hz pacing. These increases are indicative of larger cytosolic Ca^2+^ transients (not shown) and are followed by a recovery to rest levels at approximately 0.9 mN/mm^2^ during 0.25 Hz pacing. Mitochondrial Ca^2+^ levels (Figure 12C) show minimal beat-to-beat variation with significant increases in Ca^2+^ occurring gradually during periods of high pacing frequency. This property of mitochondrial Ca^2+^ loading is analogous to mitochondria acting as a low-pass filter of cytosolic Ca^2+^ changes. While the amplitude of cytosolic Ca^2+^ transients increases gradually after an increase in pacing frequency, Ca^2+^ levels always return to a diastolic value between beats. In contrast, mitochondrial Ca^2+^ accumulates almost monotonically during periods of high pacing frequency with only minor beat-to-beat variation.

**Figure 13:**
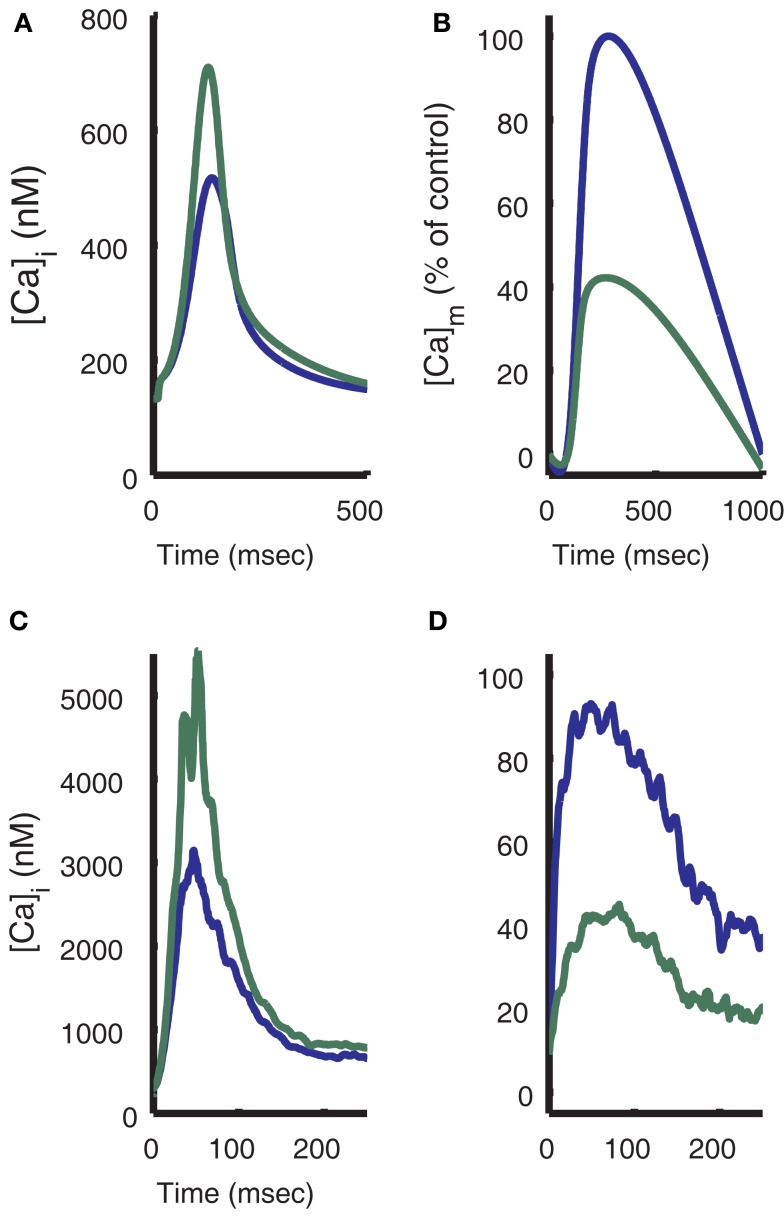
**Effect of mitochondrial uniporter block**. **(A)** Model results show the effect of 75% block of the mitochondrial uniporter (simulated by reducing the parameter Vmuni to 25% of its control value). Cytosolic Ca^2+^ transient magnitude is increased and **(B)** mitochondrial Ca^2+^ transient magnitude is decreased. Experimental data (Maack et al., 2006) for cytosolic Ca^2+^ levels **(C)** and mitochondrial Ca^2+^ levels **(D)** with addition of 10 nM Ru, a blocker of the mitochondrial uniporter.

To further illustrate the role of the Ca^2+^ uniporter in conveying cytosolic Ca^2+^ signals to the mitochondria, Figure 13 shows a comparison of model results with experimental data for uniporter block at 1 Hz pacing. After 75% of mitochondrial Ca^2+^ uniporters are blocked in the model, the cytosolic Ca^2+^ transient peak increases 51%, similar to the data shown from Maack et al. (2006). Conversely, uniporter block results in a decrease of the mitochondrial Ca^2+^ transient of about 60%. These effects also demonstrate the significance of beat-to-beat buffering of cytosolic Ca^2+^ by the mitochondria, since enough Ca^2+^ is taken up to reduce the amplitude of the cytosolic Ca^2+^ transient by approximately one-third. This also supports the results shown earlier (Figure 4) of Ca^2+^ fluxes during Ca^2+^ transient relaxation, where mitochondria exported an amount of Ca^2+^ into the cytosol 12.7% the size of the net cytosolic Ca^2+^ export flux. However, it is important to note that while the model mitochondria exchange large quantities of Ca^2+^ with the cytosol, strong buffering of Ca^2+^ in the mitochondrial matrix limits beat-to-beat variation of mitochondria Ca^2+^ transients to approximately 2% of peak mitochondrial Ca^2+^ at 1 Hz.

In this model, the influence of the mitochondria on force production and vice versa is mediated largely through changes in the concentrations of cytosolic species. As described above, the Ca^2+^ buffering properties of the mitochondria affect the amplitude of the cytosolic Ca^2+^ transient. This in turn modulates the amplitude of the force transient. The myofibrils influence the mitochondria via their influence on ADP levels. Isometric contraction is linked to ADP:ATP levels via the acto-myosin ATPase, which is responsible for the bulk of ATP hydrolysis in the contracting myocyte. Increases in workload lead to a greater consumption of ATP, which results in a rise in ADP levels. This signal is conveyed from the cytosol to the mitochondria via creatine kinase acting on the diffusible creatine phosphate pool. It is the time delay imposed by this lengthy signaling cascade that leads to the disconnect between NADH supply and subsequent complex NADH transients.

## Discussion

The work presented here describes a mathematical model which represents the electrophysiology, Ca^2+^ cycling, isometric force development and mitochondrial energetics of the guinea pig cardiac myocyte. The novel feature of this model is the incorporation of a previously developed (Hinch et al., 2004; Greenstein et al., 2006) biophysically based model of local control of SR Ca^2+^ release. An advantage of this model is that it captures the key features of CICR (gradedness of Ca^2+^ release, voltage-dependent ECC gain) and does not require computationally expensive stochastic simulations of large numbers of individual ion channels (Greenstein and Winslow, 2002; Gaur and Rudy, 2011). We have previously shown that the inclusion of such a local control mechanism, which exhibits graded SR Ca^2+^ release, is crucial for the stability of the AP (Greenstein and Winslow, 2002), given that CDI of the LCC is much stronger than VDI (Hadley and Hume, 1987; Hadley and Lederer, 1991; Sipido et al., 1995a; Linz and Meyer, 1998;Peterson et al., 1999, 2000). Without a mechanistic description of this mechanism, common pool models are unstable because the strong negative feedback on *I*_Ca,L_ via CDI resulting from regenerative RyR Ca^2+^ release into the common pool essentially switches LCC trigger flux off prematurely. While other models (Viswanathan et al., 1999; Faber and Rudy, 2000; ten Tusscher et al., 2004; Mahajan et al., 2008) have been able to reproduce experimental features of ECC by incorporating phenomenological descriptions of graded release, use of phenomenological models always presents the risk that model predictions may be less reliable. Therefore the key question is: what do we gain from the formulation of a model that incorporates a biophysically based description of local control of Ca^2+^ release? This and other issues are discussed in the following sections.

### Local control model predicts effects of AP shape on calcium-release

**Figure 14:**
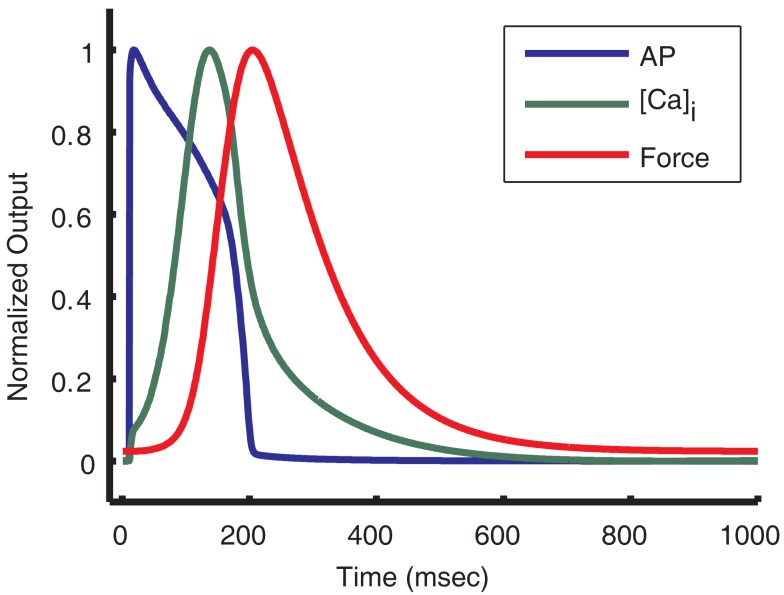
**Action potential shape causes delayed Ca^2+^ transient and force**. Normalized output from the guinea pig model is used to compare the kinetics of the AP, [Ca]_i_ transient, and force transient. As seen above, the [Ca]_i_ transient peaks near the end of the AP plateau. The force transient peak is further delayed and occurs after almost full repolarization of the cell.

The results of this study demonstrate an important functional relationship between early phase AP morphology and the kinetic properties of the cytosolic Ca^2+^ and force transients. The guinea pig ventricular myocyte model presented here, which includes an implementation of the new local control CaRU model (Greenstein et al., 2006), predicts that as a consequence of the shape of the guinea pig ventricular myocyte AP during the plateau phase, the Ca^2+^ transient peaks during the late phase of the AP (127 ms after the stimulus current; see Figure 14). This correlation of delayed Ca^2+^ transient peak with slowly repolarizing AP plateau voltage is seen in the experimental data (Arreola et al., 1991; Sah et al., 2003; Nishizawa et al., 2009). Consequently, the force transient is also delayed, having a peak that occurs after the AP is repolarized (Figure 14). This crucially important feature of the relative timing of the Ca^2+^ transient cannot be reconstructed using a common pool model, such as the prior version of the guinea pig ECME model (Cortassa et al., 2006). The delay in release timing exhibited by the present model is caused by a relatively low driving force for *I*_Ca,L_ early in the AP. While many LCCs open in the first few millisecond following the upstroke of the AP, unitary currents are small and the amount of Ca^2+^ entering any particular dyad via an open LCC is usually insufficient to trigger regenerative RyR opening. As the AP repolarizes, the driving force for *I*_Ca,L_ increases, LCC openings provide larger unitary currents, and hence are more effective at promoting the opening of RyRs, and release is more effectively triggered. This behavior, which was not built into the model, emerges as a result of incorporating the deterministic local control model which captures graded release and variable ECC gain. This model result emphasizes the role of the plateau potential in the nature of SR release triggering.

**Figure 15:**
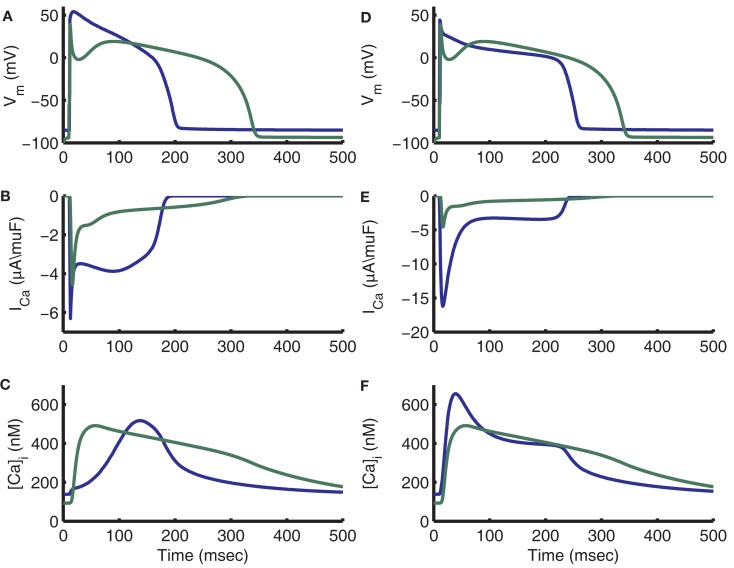
**Impact of guinea pig and canine AP morphology on *I*_Ca,L_ and [Ca]_i_ transients**. In all panels blue traces are guinea pig model output and green traces are canine model output. **(A)** Comparison of APs from guinea pig and canine models (Greenstein et al., 2006). The canine AP has a significant early repolarization notch and a significantly longer APD. **(B)** L-type current traces peak near the same value, but guinea pig shows a much larger amount of late current. **(C)** Canine [Ca]_i_ transient peak is approximately aligned with the AP notch, while the guinea pig [Ca]_i_ transient peak occurs during the late plateau phase. **(D)** On the first beat after adding *I*_to,fast_ as in the Shannon et al. (2004) model with conductance of 0.2 mS/μF, the guinea pig AP exhibits a rapid initial repolarization and APD approaches that of canine. **(E)** With addition of *I*_to,fast_, the guinea pig *I*_Ca,L_ trace exhibits a relation between fast peak and late current more similar to canine. The fast peak amplitude also increases substantially. **(F)** The peak of the guinea pig [Ca]_i_ transient is now aligned with that of the canine model.

As a consequence, it is important to note that differences in AP morphology (Figure 15A) can result in very different trigger L-Type Ca^2+^ currents (Figure 15B), and therefore Ca^2+^ transients with very different timing (Figure 15C). For example, the previously published canine myocyte model incorporating this same CaRU formulation (Greenstein et al., 2006) produced a cytosolic Ca^2+^ transient that peaked in the early phase of the AP (44 ms after the stimulus current; see Figures 15A,C). While the canine AP has a strong phase 1 repolarization due to the presence of the *I*_to_ current, guinea pig ventricular myocytes lack *I*_to_ (Findlay, 2003; Zhabyeyev et al., 2004) and their APs repolarize more slowly during the time period following the upstroke. When the guinea pig AP is at voltages near +40 mV, the canine AP has already repolarized to approximately +10 mV. As evident from the ECC gain curve (see Figure 8C), the canine AP will repolarize to a voltage at which gain is large very rapidly and trigger a relatively strong and synchronized release forming the early Ca^2+^ transient. In contrast, the slow repolarization of the guinea pig AP leads to a very slow increase in gain such that Ca^2+^ release is triggered in a more gradual manner, leading to a delay in the peak of the Ca^2+^ transient.

Many aspects of the canine and guinea pig local control models differ, including resting SR load, Ca^2+^ cycling parameters, and L-type Ca^2+^ channel parameters. To further test the hypothesis that AP shape is responsible for release timing, the control version of the guinea pig model was compared to a version with an AP which more closely resembles that of the canine model. The comparison version of the model incorporates a model of the *I*_to,fast_ current developed in the Shannon–Bers rabbit model (Shannon et al., 2004) representing the Ca^2+^-independent component of *I*_to_ (*I*_to,1_) and consisting of one Hodgkin and Huxley (1952) activation gate and a second, slower Hodgkin–Huxley inactivation gate (Figures 15D–F). Both versions of the model were started from the control model’s 1 Hz initial conditions. The added *I*_to,fast_ model introduces a brief outward current upon depolarization to positive potentials. With this current included, the AP features rapid phase 1 repolarization and a Ca^2+^ transient that triggers with much less of a delay, similar to that of the canine model (Figures 15D–F). Addition of *I*_to,fast_ hyperpolarizes the initial membrane potential of the plateau to a voltage associated with high ECC gain. After LCCs open in response to the AP upstroke to approximately +50 mV, the *I*_to_-induced repolarization reduces membrane potential and increases the driving force for *I*_Ca,L_. This larger single-channel influx of Ca^2+^ triggers a larger Ca^2+^ flux from the RyRs by way of triggering release at a larger population of release sites than for the control AP at the same time. The result is that a larger release occurs earlier in the AP with *I*_to,fast_, yielding a Ca^2+^ transient peak that occurs earlier in the AP than in control. Results were similar using an AP clamp recorded from the canine model, which yielded a Ca^2+^ transient peak of 1.05 μM occurring approximately 10 ms after the peak of the AP (not shown).

Use of a local control model such as this one featuring AP shape-dependent release will have important implications regarding behavior of tissue level model electro-mechanics. For example, in many species there are significant transmural differences in the expression levels of many key ion channels. The result is that APs from different tissue sites take on different morphologies (Nerbonne and Kass, 2005). This model predicts that these changes in AP shape will lead to changes in the timing of Ca^2+^ release, shape of the Ca^2+^ transient, and timing of force generation. AP clamp waveforms from guinea pig epicardial, midmyocardial, and endocardial cells as recorded by Sicouri et al. (1996) were input to the model to simulate the corresponding SR Ca^2+^ release and Ca^2+^ transient in each cell type (not shown). A 36% gradient of SERCa expression, measured with high resolution and accuracy by Anderson et al. (2011) was also applied. Epicardial cells were taken to have the control level of SERCa while SERCa expression was decreased by 18 and 36% in midmyocardial and endocardial cells, respectively. The resulting simulations show that release occurs earliest in midmyocardial cells due to the relatively hyperpolarized potential at the start of the AP plateau. Times to peak change by less than 10% without the application of a SERCa expression gradient, and rankings of times to peak remain the same between tissue types.

Given that this model predicts changes in release timing with AP morphology, the AP shape of the experimental species used may impact the strength of the conclusions that can be inferred about human electrophysiology. Among rabbit, canine, and human, all of which express *I*_to_, the AP notch is more prominent in recordings from epicardial than endocardial myocytes (Fedida and Giles, 1991; Liu et al., 1993; Liu and Antzelevitch, 1995; Nabauer et al., 1996). For epicardial cells, canine APs exhibit a more pronounced notch than human, while rabbit APs have a noticeable early repolarization characteristic of *I*_to_, but no strict spike-notch-dome morphology. Following phase 1 early repolarization, human AP plateau values peak below 25 mV for epicardial cells (Drouin et al., 1998; Piacentino et al., 2003). In comparison, canine plateau values for epicardial recordings at 1 Hz peak in the range of 10–15 mV (Liu et al., 1993; Liu and Antzelevitch, 1995) and rabbit plateau maxima take on values from 25 to 40 mV (Fedida and Giles, 1991; Puglisi et al., 1999). Guinea pig epicardial APs do not have a phase 1 repolarization and may take 50 ms for the plateau to decrease to 25 mV from the peak value near 50 mV (Sicouri et al., 1996). The current model predicts that these differences in notch depth and initial plateau height may significantly influence the timing of Ca^2+^ release and force generation in these different species. These results emphasize the importance of the inclusion of graded release in electromechanical models. Common pool electromechanical models without graded release are limited in their predictive scope. The all-or-none release produced by such common pool models fails to capture the sensitivity of the intracellular Ca^2+^ transient, and thus force transient, to changes in AP shape, as may occur with the reduction in *I*_to,1_ in heart failure (Greenstein et al., 2006). Some of the earliest models incorporating electrophysiology with mechanical components were limited in their predictive scope because they modeled electrophysiology without any description SR release (Li et al., 2004) or with a description resulting in all-or-none release (Li et al., 2006). However as newer release descriptions succeeded in representing graded release (Chudin et al., 1999; Shannon et al., 2004; Mahajan et al., 2008), electromechanical models incorporating those advances gained the ability to predict mechanoelectrical feedback in arrhythmia (Jie et al., 2010; Keldermann et al., 2010) and patient-specific changes in electrical activation during heart failure (Aguado-Sierra et al., 2011).

In addition to AP morphology differences between species, diseases such as heart failure can produce significant alteration of AP morphology. In human heart failure *I*_to_ density has been shown to be downregulated (Beuckelmann et al., 1993; Nabauer et al., 1996). This is the major factor in the reduction of the AP notch in recordings from isolated failing cardiac myocytes. Another consequence to AP morphology is that the initial value of the plateau is elevated in heart failure. As discussed above, the membrane potential immediately following the AP peak plays a large role in the timing and magnitude of the Ca^2+^ transient. These findings corroborate those from Greenstein and Winslow (2002), which show that different *I*_to_ current densities have a significant impact on the efficacy of ECC, making AP morphology an important factor in predicting ECC properties during heart failure.

### Critique of the model

As with any computational model, compromises must be made in order to fit the range of experimental data for different protocols. The model restitution curves for 0.5 Hz pacing shown in Figure 10A gradually reach a plateau at which APD_90_ is approximately 219 ms. The time constant for this restitution (165 ms) is much slower than that observed in experiments (Bjornstad et al., 1993; Sicouri et al., 1996). This feature of the restitution curve in the model can be attributed to the time constant of *I*_Ks_ near the resting potential. Due to the slow deactivation time constants of *I*_Ks_, there is a significant accumulation of active channels at DIs less than 1000 ms. The amount of current remains small because voltage is near the K^+^ reversal potential. If a premature stimulus shortens the DI, an even larger fraction of channels remain active, leading to larger *I*_Ks_ during the prematurely triggered AP. This results in a larger outward current that shortens the AP. Shorter DIs provide less time for *I*_Ks_ to deactivate, resulting in a larger *I*_Ks_ and shorter AP following the next stimulus. Thus the time constant of AP restitution is closely related to the time constant of *I*_Ks_ deactivation at negative potentials.

The model of *I*_Ks_ used here was formulated in the LRd99 model (Viswanathan et al., 1999) and was well fit to the two time constants of deactivation shown in experimental data (Matsuura et al., 1987). However, the Rudy group’s previous *I*_Ks_ model (Zeng et al., 1995), which shows a faster overall rate of deactivation, leads to faster restitution behavior that is a closer match to experimental data (Figures 10A,B), though still not as fast. Due to the dependence of *I*_Ks_ on the duration of diastole, this current’s kinetics affect both AP restitution and frequency-dependence behavior. The frequency-dependence of cytosolic Ca^2+^, force, and energy supply by mitochondria exhibited by the model is a good match to the experimental data (Szigligeti et al., 1996; Brandes and Bers, 1999). This model is unable to simultaneously achieve this frequency-dependent behavior and match the experimentally measured rate of AP restitution. Incorporation of a more detailed Markov model of *I*_Ks_ behavior (Silva and Rudy, 2005) may help reconcile these two cellular properties.

In addition, this model is not able to reproduce the Ca^2+^ restitution and related short-term interval-force relationships as described by Wier and Yue (1986). In other models (Rice et al., 2000; Faber et al., 2007) this behavior is mediated by a slow recovery of RyRs from inactivation. For short DIs, many RyRs remain inactivated so the maximal open probability is reduced, leading to smaller release and smaller Ca^2+^ transients. However, consistent with estimates by Bers (2001), this model has a much lower peak RyR open probability of 2.9% at 1 Hz pacing (compare to 100% for Faber et al., 2007 and 60% for Rice et al., 2000) and the proportion of channels that are closed and not inactivated does not drop below 70% at 1 Hz. The majority of RyRs are still available for activation in the event of a premature stimulus. As a result, the maximal RyR open probability triggered by a premature stimulus is not significantly reduced from control. This, coupled with the residual cytosolic Ca^2+^ stemming from the delayed Ca^2+^ transient exhibited by this model, limits the degree of Ca^2+^ restitution. A stimulus following a 1-Hz steady-state AP by a DI of 50 ms results in a premature AP with 26.5% shorter APD, but only a 7.7% smaller Ca^2+^ transient (not shown). In the coupled LCC-RyR formulation of Ca^2+^ release used in this model, JSR depletion cannot be tracked locally at individual release sites because the JSR and NSR are assumed to be in rapid equilibrium. Recent evidence suggests that the primary determinant of SR release termination and restitution of Ca^2+^ of sparks is the dynamics of local JSR refilling (Terentyev et al., 2002; Sobie et al., 2005; Zima et al., 2008b). Incorporation of a mechanistic formulation of this feature in future versions of this model should provide the ability to better reconstruct properties of Ca^2+^ restitution.

Computational models must weigh the advantages of physiological and mechanistic detail with computational efficiency in order to make useful predictions while still remaining tractable. Due to diffusion and compartmentation effects, the cardiac myocyte is subject to dynamic spatial gradients of a wide variety of ions and second messengers. In order to avoid the complexity of partial differential equations, the majority of models define compartments of uniform concentration. However the simple scenario of cytosolic, SR, mitochondrial, and dyadic compartments may not be sufficient to reproduce some experimental results. Experimental evidence (Weber et al., 2002, 2003) suggests that the concentrations of ions in close proximity to the sarcolemma may vary from those of the bulk cytosol. To account for this, several models feature a subsarcolemmal compartment (Shannon et al., 2004; Mahajan et al., 2008; Gaur and Rudy, 2011). While the model presented here was able to reproduce many critical ECC experiments without a submembrane volume, the addition of such a compartment may better account for the activity of sarcolemmal proteins that sense Na^+^ and Ca^2+^, such as NCX.

Greenstein and Winslow (2002) and Gaur and Rudy (2011) have previously published canine and guinea pig myocyte models, respectively, featuring a stochastic description of local control of SR Ca^2+^ release, a property that strongly motivated the work presented here. The ECC gain function of the low-dimensional ordinary differential equation model presented here is similar to that of the stochastic canine model. However, the ECC gain function of the Gaur and Rudy (2011) guinea pig model differs significantly from that shown here. Specifically, the gain in the model presented here decreases monotonically as membrane potential increases (Figure 8C) while the ECC gain of Gaur and Rudy (2011) decreases rapidly at hyperpolarized potentials and plateaus at a relatively high value at positive voltages. The gain for their model at +40 mV, which is the approximate peak of the AP, is roughly 10 times higher than that of the present model. As a result, Ca^2+^ release in their model (their Figure 3) is triggered fully at the start of the AP and terminates within 30 ms. In contrast, the lower gain in the present model at membrane potentials near the peak of the AP leads to incomplete, but persistent release that inactivates gradually during phase 3 repolarization. While, to the authors’ knowledge, voltage-dependent ECC gain has not been measured in guinea pig, the relative displacement of voltage-dependent LCC and RyR fluxes in this model closely resemble the shifted bell-shaped curves that give rise to the monotonic decreasing voltage-dependent ECC gain function measured in rat (Wier et al., 1994; Santana et al., 1996; Song et al., 2001).

An alternative approach to modeling graded release in deterministic myocyte models is to utilize more abstracted release descriptions. For example, the Rudy group (Faber et al., 2007; Gaur and Rudy, 2011) modeled the rate of Ca^2+^ flux through open RyRs as a function of the voltage-dependent Ca^2+^ trigger flux. Mahajan et al. (2008) utilized a simpler formulation lacking any description of RyR gating. Instead, the rate of Ca^2+^ release events was modeled as being proportional to the product of LCC open probability and a voltage-dependent gain function. One advantage of such models formulations is that they “break” the strong positive feedback loop that is well known to lead to all-or-none Ca^2+^ release in common pool models (Stern, 1992). These formulations can also reduce the number of model state variables, facilitating large-scale simulations at the tissue and whole-heart levels. In fact the Mahajan model (2008) is able to reproduce the late peak of the Ca^2+^ transient observed in this work through its phenomenological incorporation of voltage-dependent gain. However, a disadvantage of these more abstract models is that they cannot be used to predict the effects of events such as fundamental changes in RyR gating on ECC gain properties (Hashambhoy et al., 2010) without additional assumptions, because the gain function itself is built directly into the models.

The approach of approximating cellular function using more abstract (i.e., less mechanistic) descriptions to reduce the number of state variables could also be applied to the mitochondria. In a scenario where abnormal ATP supply does not need to be addressed, the Ca^2+^ buffering role of the mitochondria could be approximated by a slow buffer. Such an approximation would continue to influence the cytosolic Ca^2+^ transients on a beat-to-beat basis and over longer periods of stimulation by accumulating or releasing Ca^2+^ slowly as cytosolic Ca^2+^ peaks change. However, the slow buffer approximation would begin to break down under conditions where a large mitochondrial to cytosolic Ca^2+^ gradient is present, such as upon commencement of beta adrenergic stimulation.

The model presented here lacks a JSR compartment, which was combined with the network SR in developing the deterministic formulation of the CaRU model (Hinch et al., 2004). As a result, the model does not reproduce local SR depletion during release. Instead, this CaRU model relies on RyR inactivation for termination of release. While some experimental data supports inactivation as a possible mechanism of release termination (Sham et al., 1998), more recent work suggest that local JSR Ca^2+^ depletion may be the major mechanism for release termination (Terentyev et al., 2002; Zima et al., 2008a,b; Domeier et al., 2009; Stevens et al., 2009). The RyR model presented here precludes the possibility of incorporating local JSR depletion. In addition, other groups (Shannon et al., 2004; Mahajan et al., 2008; Gaur and Rudy, 2011) have incorporated SR load-dependence into RyR behavior, a feature not included in this model. Its inclusion may help reconstruct the steep SR load-dependent increase in fractional release seen in some datasets (Shannon et al., 2000).

Other models that incorporate both ECC and mitochondrial energetics exist. Magnus and Keizer (1998) developed one of the first comprehensive models of cytosolic and mitochondrial Ca^2+^ handling in pancreatic β-cells. Their Ca^2+^-regulation of the TCA cycle was the basis on which the energetics model used here was developed (Cortassa et al., 2003, 2006). Subsequently, Matsuoka et al. (2004) formulated a cardiac myocyte model describing electrophysiology and Ca^2+^ handling between mitochondria and cytosol. However, this model is not able to capture the biphasic response of NADH levels to changes in pacing frequency. Another recent model (Hatano et al., 2011) is novel in that it describes electrophysiology, mitochondrial energetics, and spatiotemporal changes in Ca^2+^ and many metabolites. While this model supports the existence of subsarcolemmal Ca^2+^ gradients as predicted by NCX calculations (Weber et al., 2002), and predicts the cellular distribution of ADP, the coarse resolution of the 3D mesh required for computational tractability precludes the ability to make predictions regarding subspace Ca^2+^ levels. The advantage of the model presented here is that its mechanistic description of ECC and Ca^2+^ cycling in the cell will provide a platform for future investigations in Ca^2+^-regulation of mitochondrial energy production.

## Conclusion

We have developed a mechanistically detailed description of ECC in the guinea pig cardiac myocyte combined with modules describing energetics and isometric force. This model successfully reproduces key ECC properties of graded SR Ca^2+^ release and voltage-dependent gain. Additionally, the incorporation of mitochondrial energetics allows the model to qualitatively reproduce changes in NADH in response to changes in cardiac workload. Using this model we can improve our understanding of how changes in AP shape and Ca^2+^ transients affect energy supply and developed force in normal and failing myocytes.

## Conflict of Interest Statement

The authors declare that the research was conducted in the absence of any commercial or financial relationships that could be construed as a potential conflict of interest.
